# Emissions of Semi-Volatile Organic Compounds from Architectural Coatings and Polyvinyl Chloride Floorings: Microchamber Method

**DOI:** 10.3390/molecules29184445

**Published:** 2024-09-19

**Authors:** Hongyan Guan, Qi Jia, Zhongbao Guo, Xu Han, Huiyu Zhang, Liteng Hao, Chuandong Wu, Jiemin Liu

**Affiliations:** 1 China Testing & Certification International Group Co., Ltd., Beijing 100024, China; guanhongyan@ctc.ac.cn (H.G.); jiaqi@ctc.ac.cn (Q.J.); zhanghuiyu@ctc.ac.cn (H.Z.); haoliteng@ctc.ac.cn (L.H.); 2 School of Chemistry and Biological Engineering, University of Science and Technology Beijing, Beijing 100083, China; hx2468013579@163.com (X.H.); wuchuandong@ustb.edu.cn (C.W.); liujm@ustb.edu.cn (J.L.); 3 Beijing Institute of Graphic Communication, Beijing 102600, China

**Keywords:** semi-volatile organic compounds (SVOCs), building materials, emission characterization, microchamber method, polyvinyl chloride (PVC)

## Abstract

Semi-volatile organic compounds (SVOCs) are modern chemical substances that are present in large quantities in indoor environments. Understanding the emission of SVOCs from building materials is essential to identify the main sources of indoor SVOCs and to improve indoor air quality. In this study, a reference method employing custom-designed microchambers (630 mL) was optimized by improving the structure of the gas path and adding polytetrafluoroethylene inner coating to the chamber. After optimization, the recoveries of the microchamber method were significantly improved (75.4–96.7%), and the background in the microchamber was greatly reduced (<0.02 μg/h). By using the microchamber method, 33 SVOCs (including two alkanes, one aromatic, one nitrogen compound, and twenty-nine oxygenated compounds) and 32 SVOCs (including seven alkanes, eight aromatics, and seventeen oxygenated compounds) were detected in the emissions of the architectural coating and the PVC flooring samples, respectively. The area-specific emission rates (*SER*_a_) of total SVOCs emitted from architectural coatings and PVC floorings were in the range of 4.09–1309 μg/m^2^/h) (median: 10.3 μg/m^2^/h) and 0.508–345 μg/m^2^/h (median: 11.9 μg/m^2^/h), respectively. Propanoic acid had the highest *SER*_a_ (3143 μg/m^2^/h) in architectural coatings, while methylbenzene (345 μg/m^2^/h), 2-methylnaphthalene (65.2 μg/m^2^/h), and naphthalene (60.3 μg/m^2^/h) were main SVOCs emitted from PVC floorings. Meanwhile, the average second-stage (adsorbed phase) emission mass of the total SVOCs accounts for 66.3% and 47.3% in architectural coatings and PVC floorings, respectively, suggesting that the SVOCs emitted from building materials have a strong tendency to be absorbed on the surface of the room, e.g., the interior wall, the desk or even the skin.

## 1. Introduction

With the increasing level of urbanization, a significant amount of building materials are utilized for interior decoration [1,2]. Building materials release various semi-volatile organic compounds (SVOCs) during construction and application, which serve as the primary source of indoor air pollution [3]. SVOCs are ubiquitous indoors due to the widespread use of building materials [4,5,6,7]. Because of their low vapor pressure, SVOCs tend to be redistributed through indoor air from their original sources to other solid phases, including skin, clothing, airborne particles, and dust. Human exposure to certain SVOCs has been associated with adverse health effects, e.g., asthma, allergies, and reproductive abnormalities [8,9,10]. Moreover, some SVOCs are recognized as potential endocrine disruptors [11,12]. In general, most people spend most of their time indoors: 20 h/day for adults and 21 h/day for children in China. Long-term exposure to SVOCs can lead to adverse effects such as Sick Building Syndrome (SBS) [13,14,15]. Therefore, exposure to indoor SVOCs is a matter of great concern [16].

The emission of SVOCs from source materials usually occurs slowly, and the gas-phase SVOCs are easily adsorbed by internal surfaces, suspended particles, and settled dust. Because of the low concentration of SVOCs in the gas phase, strong adsorption to solid surfaces, ubiquitous contamination in laboratory equipment, and complex sampling and analysis procedures, there are few studies on the measurement of SVOC emissions. Emission chambers are presently the primary tool for measuring SVOC emissions from typical sources [17]. Kemmlein et al. [18] investigated various building materials and consumer goods (e.g., insulating materials, assembly foam, upholstery/mattresses, and electronics equipment) for emissions of Tris (2-chloro-isopropyl)phosphate (TCIPP), hexabromocyclododecane (HBCD), tetrabromobisphenol A (TBBPA), tris(2-chloroethyl) phosphate (TCEP), etc. Three types of emission test chambers (two glass cells with volumes of 0.001 m^3^ and 0.02 m^3^ and one standard emission stainless steel test chamber with a volume of 1 m^3^) were used. TCIPP was one of the most frequently emitted SVOCs in test samples, with the controlled emission chamber conditions of 23 ± 0.1 °C and 50 ± 3% RH, the area-specific emission rates (*SER*_a_) of TCIPP varied from 20 ng/m^2^/h (upholstery stool) to 140 μg/m^2^/h (assembly foam). However, after a test period of more than 100 days, no emissions of HBCD from insulating boards were detected in the air. This may be attributed to the high experimental limits of detection (0.09–1.8 ng/m³) and the low sampling volumes (5–40 m³). A similar issue with SVOCs was also reported by Bakó-Biró et al. [19]. For SVOCs, the adsorption by the chamber walls is inevitable, which may introduce errors in the measurement of emissions [20,21,22,23]. In addition, the low emission concentration of SVOCs requires longer sampling periods and larger sampling volumes. As a result, the development of appropriate methods to measure the emissions of SVOCs from various sources has become a high priority and the creation of specially designed chambers has been a key prerequisite for measuring SVOCs. A novel micro-chamber method has been developed to determine the emission of SVOCs in our previous studies [21,23]. Compared with the traditional environmental chamber method, the microchamber method could measure the emitted SVOCs from materials at both the gaseous phase and the adsorbed phase by thermal desorption reducing the sink effect of the SVOCs. Also, the microchamber can shorten the time to reach steady state by increasing the ratio of emission surface to sorption surface.

Architectural coatings and polyvinyl chloride (PVC) floorings are commonly used in interiors (e.g., homes, offices, and dormitories) for their low cost, aesthetic, and serviceable characteristics [24,25,26]. Notably, a broad range of additives are used in coatings and PVC floorings to improve their adhesion, durability, stability, flame retardant, etc. [27,28,29]. For example, methylbenzene and xylene are commonly used as solvents and curing agents in architectural coatings, aiding in the long-lasting dissolution of other substances in the coatings [30]. Additionally, they help prevent issues such as cracking and peeling [31], and 2-methylnaphthalene and naphthalene are common plasticizers in the production of PVC materials [32,33]. It has been demonstrated that these SVOCs can transfer from materials into indoor air during usage, leading to a significant decline in the quality of indoor air [34,35,36,37]. Therefore, it is necessary to accurately identify the main SVOCs emitted from indoor building materials and examine their emission characteristics.

The primary objectives of this study were to (1) optimize the microchamber test system and evaluate the microchamber method; (2) apply the microchamber method to identify the main SVOC pollutants released from architectural coatings and PVC floorings; (3) investigate the emission characteristics of SVOCs in different building materials, and to further enhance our understanding of the impact of these building materials on indoor air pollution.

## 2. Results and Discussion

### 2.1. The Background in the Microchamber

**Figure 1:**
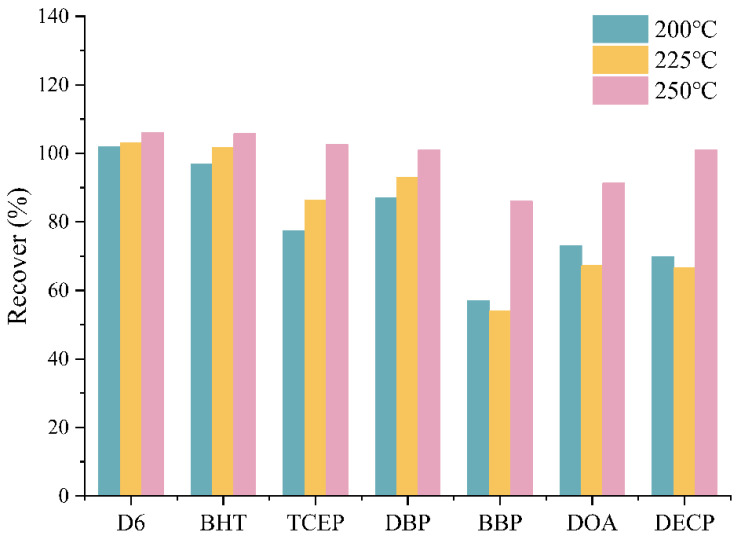
Recoveries of target SVOCs by microchamber method at different thermal desorption temperatures.

Evaluation of the background concentration and recovery rate of the optimized microchamber was conducted in this study, and experimental results demonstrated that the chamber could meet the requirements of SVOC testing in the standard. Before testing, the chamber interior and lid were wiped with ethanol and allowed to air dry naturally, followed by purging with inert gas, slow heating up to 250 °C, and blowing at high temperature for 4–5 h. Air samples blown out from the microchamber were collected for background testing. The chromatogram of the background SVOC in the microchamber after ethanol clean-up is presented in Figure 1. The results showed that the SVOC content of each substance in the microchamber was below 0.02 μg within 1 h, indicating that the microchamber method met the requirements of SVOC testing in GB/T 42898-2023 [38].

### 2.2. Recoveries for Typical SVOCs in the Microchamber Method

#### 2.2.1. Comparison of SVOC Recoveries at Different Thermal Desorption Temperatures

The recoveries of the optimized microchamber were evaluated using 7 SVOCs, including typical SVOCs found in building materials such as antioxidants, flame retardants, and plasticizers (Table 1). Data analysis revealed that low-boiling point substances such as D6 and BHT showed minimal variations in recovery rates at different temperatures (Figure 1). High-boiling point substances exhibited relatively higher recovery rates at 250 °C, reaching approximately 80%. Temperatures of 250 °C or higher should be preferred for testing the release of high boiling point aromatic SVOCs.

#### 2.2.2. Comparison of SVOC Recoveries in Different Microchambers

**Figure 2:**
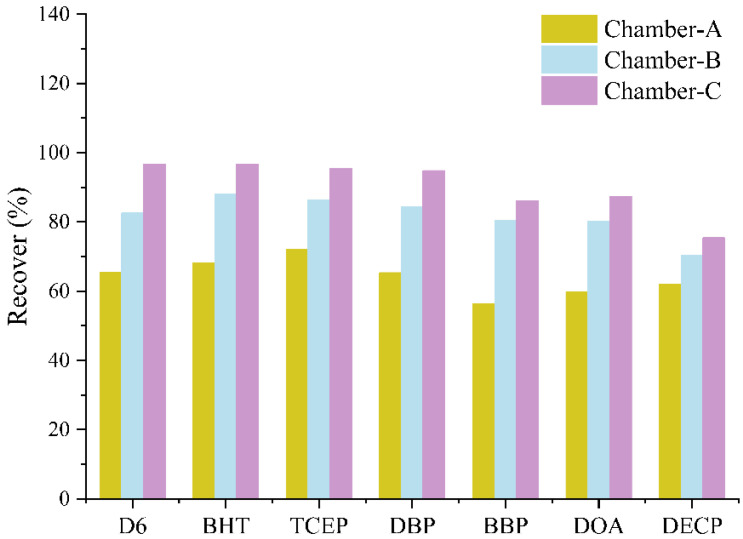
Recoveries of target SVOCs by microchamber method in different chambers. Chamber A: the microchamber before optimization; Chamber B: the first-generation optimization chamber; Chamber C: the second-generation optimization chamber.

The microchamber structure has been optimized, with the optimization process detailed in Section 3.2. Figure 2 illustrates a comparison of recoveries of target SVOCs in various chambers. The recoveries of target SVOCs using the microchamber method in the original chamber, the first-generation optimized chamber, and the second-generation optimized chamber were in the range of 56.4–72.1%, 70.4–88.0%, and 75.4–96.7%, respectively. The results indicate that after optimization, the recoveries of the microchamber method were significantly improved, and the deposition of target contaminants in the bulkhead and pipelines was greatly reduced, which is conducive to improving test accuracy.

### 2.3. Quality Assurance and Quality Control

#### 2.3.1. Establishment of SVOC Test Calibration Curves

Standard curves were constructed using standard solutions of the selected target compounds. Specifically, 0.1000 g of D6, BHT, TCEP, DBP, BBP, DOA, and DEHP were individually weighed into 100 mL volumetric flasks and then diluted to volume with chromatography-grade acetone to prepare standard stock solutions. These stock solutions were diluted with acetone to obtain mixed standard solutions with concentrations of 0.1, 0.5, 1, 2, 5, 7, and 10 mg/L. GC/MS was used to analyze the mixed standard solutions. The standard curves were generated by plotting the peak area response values against the mass of the target compounds (µg), and strong linearity (*R*^2^ > 0.999) was achieved. Details on calibration curves and their linear correlation coefficient for the target compounds are listed in Table 2.

#### 2.3.2. Method Limits of Detections

The standard sample with the lowest concentration was replicated seven times, and the mass of each compound was determined using the standard curve and the standard deviation (SD) calculation. The Method limits of detections (MDLs) were calculated as three times the SD of the procedural blank values plus the average procedural blank levels. Further details are listed in Table 3.

#### 2.3.3. Method Precision and Sample Recovery

The actual sample with a certain content was precisely added to the blank adsorption tube and analyzed according to the sample analysis steps. Each sample was measured six times in parallel, and the relative standard deviation and standard recovery rate were determined. The results, presented in Table 4, revealed recoveries ranging from 90.6% to 119% and relative standard deviations ranging from 4.03% to 13.7%, indicating that the method has high recovery and precision for the analysis of SVOCs.

### 2.4. Application of the Microchamber Method

#### 2.4.1. SVOC Emissions from Each Architectural Coating

**Figure 3:**
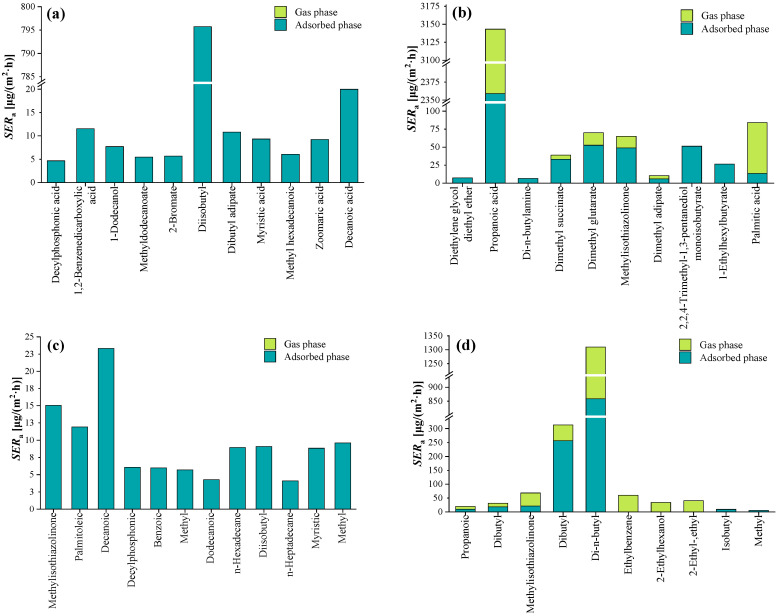
Gas-phase and adsorbed-phase chamber concentrations of SVOCs emitted from architectural coatings. (**a**) Coating sample A; (**b**) coating sample B; (**c**) coating sample C; (**d**) coating sample D.

Following a comparative analysis of chromatographic peaks and the elimination of blank interference, the release results of SVOCs for each architectural coating sample were obtained and presented in Figure 3. The emission characteristics of SVOCs exhibit heterogeneities in different coating samples. In total, 33 SVOCs were detected emitted from the four architectural coatings using the emission chamber: two alkanes (n-hexadecane, n-heptadecane), one aromatic (ethylbenzene), one nitrogen compound (di-n-butylamine), and twenty-nine oxygenated compounds. The *SER*_a_ of total semi-volatile organic compounds (TSVOC) emitted by architectural coatings ranged from 4.09 μg/(m^2^·h) to 1309 μg/(m^2^·h), with a median of 10.3 μg/(m^2^·h). Propanoic acid and di-n-butyl glutarate had the highest emission rate (3143 and 1309 μg/(m^2^·h), respectively). Many studies have reported the presence of propanoic acid and di-n-butyl glutarate in indoor environments [39,40,41,42], but little information is available on its sources. This study suggested that one of the sources of propanoic acid and di-n-butyl glutarate in indoor environments might be SVOCs emitted by architectural coating.

The SVOCs emitted from the coating samples A and C were all absorbed into the chamber’s inner surface. On average, the emitted SVOCs in the gas phase account for only 33.7% of the total mass, which suggests that the SVOCs emitted from the architectural coating were primarily absorbed on the surface of the room, e.g., the interior wall, the desk, or even the skin. Because most SVOCs have low vapor pressures. For example, the vapor pressure of diisobutyl adipate is as low as 1.75 × 10^−7^ Pa at 25 °C, which means that diisobutyl adipate has a strong tendency to adsorb on surfaces.

#### 2.4.2. SVOC Emissions from Each PVC Flooring

32 SVOCs were detected emitted from the three PVC floorings using the emission chamber: seven alkanes (n-hendecane, tritriacontane, n-heptadecane, n-hexadecane, n-heneicosane, dodecane, octamethyl cyclotetrasiloxane), eight aromatics (styrene, methylbenzene, naphthalene, α-methylnaphthalene, 2-methylnaphthalene, 2-ethynyl naphthalene, 1,3-dimethyl naphthalene, 1,5-dimethyl naphthalene), and seventeen oxygenated compounds (1-methoxy-2-propyl acetate, 2-ethyl hexanol, benzoic acid, 2-hydroxy-2-methylpropiophenone, Isobutyl benzoate, methyl Laurate, benzophenone, 4-chlorobenzophenone, di-iso-butyl phthalate, methyl 2-benzoyl benzoate, palmitic acid, dibutyl Phthalate, di(2-ethylhexyl)phthalate, diethylene glycol dibutyl ether, methyl palmitate, levoglucosenone, and 1-decane phosphonic acid).

**Figure 4:**
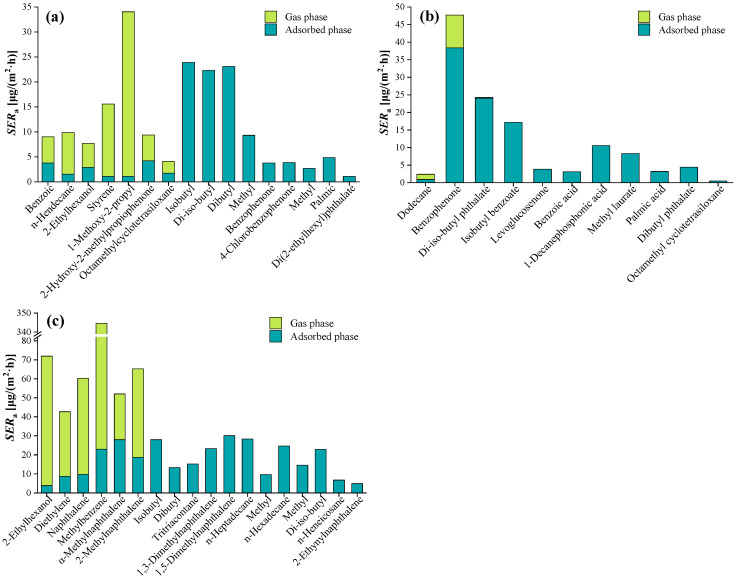
Gas-phase and adsorbed-phase chamber concentrations of SVOCs emitted from PVC floorings. (**a**) Flooring sample A; (**b**) flooring sample B; (**c**) flooring sample C.

Figure 4 shows the *SER*_a_ of each compound emitted from the PVC flooring samples. The *SER*_a_ of TSVOC emitted by PVC floorings ranged from 0.508 μg/(m^2^·h) to 345 μg/(m^2^·h), with a median of 11.9 μg/(m^2^·h). The top three SVOCs in emission rate were methylbenzene (345 μg/(m^2^·h)), 2-methylnaphthalene (65.2 μg/(m^2^·h)), and naphthalene (60.3 μg/(m^2^·h)). This is consistent with previous studies, which also detected methylbenzene, 2-methylnaphthalene, and naphthalene in indoor air [43,44,45]. SVOCs in the adsorbed phase account for 47.3% of the total mass on average. The partition coefficient (ratio of the released mass of SVOCs in the chamber’s gaseous phase to that in the adsorbed phase) of the 32 detected SVOCs was in the range of 0−30.0, indicating that SVOCs emitted from the PVC floorings have a strong tendency to adsorb on surfaces.

## 3. Materials and Methods

### 3.1. Test Pieces

Seven different interior building materials, including four architectural coatings and three polyvinyl chloride (PVC) floorings, were collected from a construction materials plant in China according to their popularity. These samples are representative products of different brands in the Chinese domestic market and have different properties or prices. Each architectural coating sample was applied on a 50 cm^2^ stainless steel sheet and cured for 48 h at a temperature of 23 °C and a humidity of 50 ± 10%. Each PVC flooring sample was cut into 50 cm^2^ sheets at randomly chosen positions, wrapped in aluminum foil, and also aged for 48 h under the same conditions. Prior to a measurement, the test pieces were unpacked and placed in the emission chamber.

### 3.2. Emission Chamber

**Figure 5:**
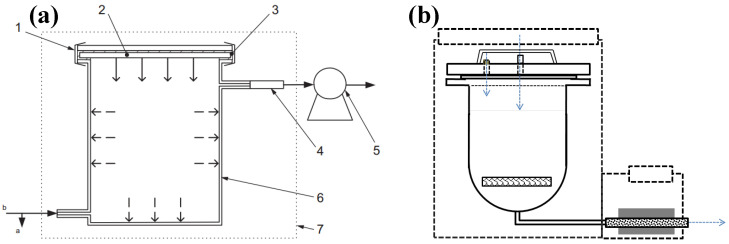
Schematic of the microchamber before (**a**) and after (**b**) the optimization. (1: fixture; 2: sample; 3: sealing material; 4: adsorbent tube (air sampling in microchamber); 5: sampling pump; 6: microchamber; 7: incubator); Arrows indicate the direction of carrier gas flow.

The design of the microchamber used in previous experiments was improved. The schematics of the microchamber before and after optimization are shown in Figure 5 [21,46]. The part and reason for the microchamber optimization are listed in Table 5. The first-generation microchamber (Figure 5a) was initially designed with two microchambers, which could simultaneously conduct the emission test on two samples. The chamber utilized a glass rotor flowmeter to control the gas flow rate, and the inner wall of the chamber was made of mirror stainless steel. However, problems with the flow meter and seals resulted in elevated background concentrations within the chamber. In contrast, problems with the gas inlet and chamber lid seals resulted in SVOC recovery rates of 50–60%. Therefore, we customized the second-generation microchamber (Figure 5b) [21,46]. To reduce the sink effect, the microchamber was mainly reformed in two aspects. On the one hand, the structure of the gas path was improved so that N_2_ could enter the chamber directly without passing through the flowmeter. On the other hand, the inner wall and sealing cover of the chamber were coated with polytetrafluoroethylene to reduce the adsorption to the inner wall. Meanwhile, in order to improve the tightness of the chamber system, we replaced the seals and the electronic flowmeter.

### 3.3. Emission and Gas Sampling

**Figure 6:**
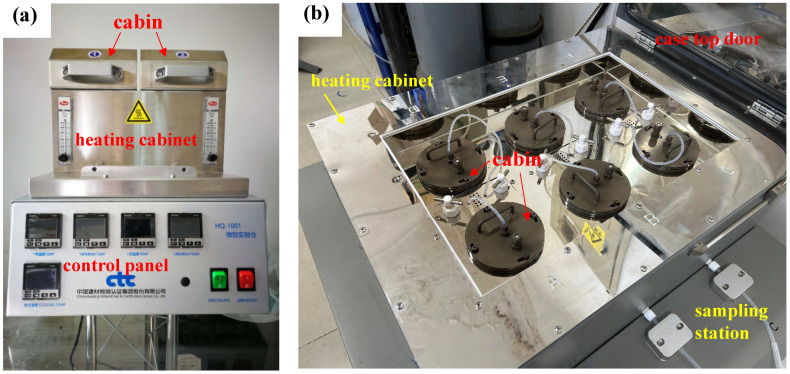
Physical picture of the optimized microchamber. (**a**) The first generation; (**b**) the second generation.

This study uses a microchamber (630 mL, inner chamber: inert coated, stainless steel) (Figure 6) through a set of testing conditions (environmental temperature, relative humidity, and ventilation) and sampling conditions (Table 6) according to ISO 16000-25:2011 [47]. Apparatus preparation included thorough cleaning of the microchamber using methanol, acetone, and ethyl acetate in sequence, as well as testing of the microchamber background. High-purity nitrogen was introduced into the chambers with an outlet flow rate of 15 mL/min, and the gas flow rates were controlled by an electronic flowmeter. The emission samples were actively sampled on Tenax TA adsorbent tubes referred to ISO 16000-25:2011 [47], and the tube was affixed to the outlet to capture SVOCs in the gas phase over 24 h. After a 24-hour sampling of SVOCs from architectural coatings and PVC floorings, then, the test piece was taken out, the temperature increased to 250 °C, and the SVOCs adsorbed on the wall surface were desorbed and sampled. The test method was obtained by a two-stage time-dependent determination of emission test (first step test, gaseous phase) and heating-up desorption test (second step test, absorbed phase).

The concentration of each component in the emission samples should be calculated according to Equation (1).

(1) $$C_{i}=\frac{{m_{i}-m}_{0i}}{V}$$

where $C_{i}$ is the concentration of the emitted SVOCs in the microchamber (ng/m^3^); $m_{i}$ is the mass of the SVOCs in the sample sorbent tube (ng); $m_{oi}$ is the mass of the SVOCs in the blank sorbent tube (ng); *V* is the volume of air sampling (m^3^).

The ${SER}_{a}$ of each SVOC in the specimen is calculated according to Equation (2).

(2) $${SER}_{a}=\frac{m_{1}+m_{2}}{At}$$

where $m_{1}$ is the mass collected in the emission test (first step test) (ng); $m_{2}$ is the mass collected in the absorption test (second step test) (ng); A is the surface area of the test specimen (m^2^); t is the duration of the first phase (h).

### 3.4. Analysis Method

The SVOCs collected on the Tenax TA tubes were analyzed using a thermal desorber combined with gas chromatography coupled to mass spectrometry (TD100-XR, Marks (Calgary, Canada), desorb temperature: 300 °C, desorb time: 10 min, trap flow: 30 mL/min, trap low temperature: −10 °C, trap high temperature: 300 °C, flow path temperature: 250 °C, S/SL mode: splitless). All samples were analyzed using a GC-MS (QP2020, Shimadzu (Kyoto, Japan)) in the SCAN mode and electron ionization (EI). Separation was conducted on a column (DB-5MS, length 30 m, internal diameter 0.25 mm, film thickness 0.25 μm) using a thermal gradient: 50 °C for 2 min, 20 °C/min to 200 °C, held for 8 min, 8 °C/min to 300 °C for 12 min. The injector temperature was 300 °C with helium as carrier gas at 8 mL/min.

## 4. Conclusions

In this study, the microchamber was improved by modifying the gas path and adding polytetrafluoroethylene coating to reduce the SVOCs’ sink effects, i.e., sorption to chamber components. After optimization, the background concentration of a single SVOC did not exceed 0.02 μg within 1 h, and the recoveries of SVOCs during the whole emission test were in the range of 90.6–119% (SD: 4.03–13.7%), which met the requirements in GB/T 42898-2023 [38]. The SVOCs emitted from architectural coatings and PVC floorings were analyzed by the microchamber methods. The results indicated that the SVOCs emitted from architectural coatings with the highest emission rates were propanoic acid and di-n-butyl glutarate, and the SVOCs emitted from PVC floorings with the highest emission rates were methylbenzene, 2-methylnaphthalene, and naphthalene. The two-stage emission rates are different. On average, the second-stage (adsorbed phase) emission mass of SVOCs from the architectural coating and PVC floorings account for 66.3% and 47.3% of the total mass, respectively, meaning the emitted SVOCs have a strong tendency to adsorb on surfaces. This comprehensive research into chemical emissions could help rapidly identify indoor sources of SVOCs, identify the risks associated with SVOCs in building materials, and prioritize a range of chemicals of concern to SVOCs based on risk.

## Figures and Tables

**Table 1:** Typical SVOCs for evaluation.

Abbreviation	Name	CAS#	Application
D6	Dodecamethylcyclohexasiloxane	540-97-6	softening agent
BHT	Butylated hydroxytoluene	128-37-0	antioxidant
TCEP	Tris(2-chloroethyl) phosphate	115-96-8	flame retardant
DBP	Dibutyl phthalate	84-74-2	plasticizer
DOA	Bis(2-ethylhexyl) adipate	103-23-1	plasticizer
DEHP	Dioctyl Phthalate	117-81-7	plasticizer
BBP	Butyl Benzyl Phthalate	85-68-7	plasticizer

**Table 2:** Calibration curves for the target SVOCs in the emission test.

Analytes	Calibration Curves	*R* ^2^
D6	y = 510249x + 22982	0.9999
BHT	y = 2966456x − 183151	0.9991
TCEP	y = 897991x − 57186	0.9983
DBP	y = 6448849x − 255305	0.9998
BBP	y = 2597967x − 58986	0.9998
DOA	y = 2860857x − 44271	0.9996
DEHP	y = 3746605x − 75712	0.9997

**Table 3:** Method limits of detections (MDLs) for the target SVOCs in the microchamber emission test.

Analytes	Mass of Emission (μg)								SD (%)	MDLs (μg)
	Test 1	Test 2	Test 3	Test 4	Test 5	Test 6	Test 7	Mean		
D6	0.023	0.026	0.029	0.027	0.026	0.022	0.026	0.026	0.002	0.006
BHT	0.013	0.016	0.015	0.020	0.016	0.016	0.016	0.015	0.001	0.004
TCEP	0.018	0.019	0.018	0.024	0.018	0.021	0.020	0.019	0.001	0.003
DBP	0.025	0.024	0.022	0.022	0.024	0.028	0.023	0.024	0.002	0.005
BBP	0.023	0.023	0.021	0.025	0.023	0.025	0.024	0.023	0.001	0.004
DOA	0.024	0.026	0.022	0.025	0.027	0.027	0.025	0.025	0.002	0.005
DEHP	0.021	0.027	0.018	0.025	0.027	0.025	0.028	0.024	0.004	0.011

**Table 4:** Method precision and recoveries.

Analytes	Mass of Emission (μg)						RSD (%)	Recoveries (%)
	Test 1	Test 2	Test 3	Test 4	Test 5	Test 6		
D6	0.748	0.580	0.616	0.565	0.546	0.520	13.7	119
BHT	0.533	0.511	0.494	0.481	0.484	0.486	4.03	99.6
TCEP	0.535	0.474	0.438	0.401	0.418	0.451	10.6	90.6
DBP	0.573	0.503	0.475	0.463	0.468	0.505	8.17	99.6
BBP	0.583	0.512	0.481	0.480	0.488	0.527	7.72	102
DOA	0.587	0.531	0.502	0.497	0.502	0.543	6.55	105
DEHP	0.573	0.512	0.481	0.480	0.484	0.522	7.10	102

**Table 5:** Part and reason of the microchamber optimization.

Before the Optimization	After the Optimization	Reason
Sample clamped between hatch cover and hatch body.	Samples are stored on hold or on a sample rack.	Solid flaky samples should be cut into round flaky samples before optimization. After optimization, it is suitable for testing thicker and deformed samples.
The air intake was located on the lower side of the chamber.	The air intake was located on the cover of the chamber.	To reduce the SVOC deposition in the chamber.
The bottom of the chamber was flat.	The bottom of the chamber was streamlined.	To reduce the SVOC deposition in the chamber.
No cooling device.	The electronic cooling unit was added for gas sampling at low temperatures.	To improve the capture efficiency of the SVOC.

**Table 6:** Measurement conditions of the two-stage microchamber emission test.

First Emission Stage	
Gas supply	N_2_ (0.9 L/h)
Temperature and humidity	23 ± 0.5 °C and 50 ± 5% RH
Sampling rate	21.6 L (15 mL/min × 24 h)
Scavenger	Tenax TA
**Second Thermal Desorption Stage**	
Gas supply	N_2_ (5.4 L/h)
Temperature of the thermal desorption system	23 °C → 15 °C/min → 250 °C
Sampling rate	3.6 L (90 mL/min × 40 min)
Scavenger	Tenax TA

## Data Availability

Data will be made available upon reasonable request.
